# Broadly Inhibiting Antineuraminidase Monoclonal Antibodies Induced by Trivalent Influenza Vaccine and H7N9 Infection in Humans

**DOI:** 10.1128/JVI.01182-19

**Published:** 2020-01-31

**Authors:** Pramila Rijal, Bei Bei Wang, Tiong Kit Tan, Lisa Schimanski, Philipp Janesch, Tao Dong, John W. McCauley, Rodney S. Daniels, Alain R. Townsend, Kuan-Ying A. Huang

**Affiliations:** a Center for Translational Immunology, Chinese Academy of Medical Sciences Oxford Institute, Nuffield Department of Medicine, University of Oxford, Oxford, United Kingdom; b MRC Human Immunology Unit, MRC Weatherall Institute of Molecular Medicine, Radcliffe Department of Medicine, University of Oxford, Oxford, United Kingdom; c Institute of Infectious Diseases, Beijing Ditan Hospital, Capital Medical University, Beijing, China; d Worldwide Influenza Centre, The Francis Crick Institute, London, United Kingdom; e Division of Infectious Diseases, Department of Paediatrics, Chang Gung Memorial Hospital, Taoyuan, Taiwan; f School of Medicine, Chang Gung University, Taoyuan, Taiwan; St. Jude Children's Research Hospital

**Keywords:** monoclonal antibodies, influenza virus, ELLA, H7N9 virus, influenza neuraminidase, immune response, immunization

## Abstract

The majority of antibodies induced by influenza neuraminidase (NA), like those against hemagglutinin (HA), are relatively specific to viruses isolated within a limited time window, as seen in serological studies and the analysis of many murine monoclonal antibodies (MAbs). We report three broadly reactive human MAbs targeting N1 NA. Two were isolated from a young adult vaccinated with trivalent influenza vaccine (TIV), which inhibited N1 NA from viruses isolated from humans over a period of a hundred years. The third antibody, isolated from a child with acute mild H7N9 infection, inhibited both group 1 N1 and group 2 N9 NAs. In addition, the antibodies cross-inhibited the N1 NAs of highly pathogenic avian H5N1 influenza viruses. These antibodies are protective in prophylaxis against seasonal H1N1 viruses in mice. This study demonstrates that human antibodies to N1 NA with exceptional cross-reactivity can be recalled by vaccination and highlights the importance of standardizing the NA antigen in seasonal vaccines to offer optimal protection.

**IMPORTANCE** Antibodies to the influenza virus NA can provide protection against influenza disease. Analysis of human antibodies to NA lags behind that of antibodies to HA. We show that human monoclonal antibodies against NA induced by vaccination and infection can be very broadly reactive, with the ability to inhibit a wide spectrum of N1 NAs on viruses isolated between 1918 and 2018. This suggests that antibodies to NA may be a useful therapy and that the efficacy of influenza vaccines could be enhanced by ensuring the appropriate content of NA antigen.

## Abstract (precis)

Antibodies to the influenza virus NA can provide protection against influenza disease. Analysis of human antibodies to NA lags behind that of antibodies to HA. We show that human monoclonal antibodies against NA induced by vaccination and infection can be very broadly reactive, with the ability to inhibit a wide spectrum of N1 NAs on viruses isolated between 1918 and 2018. This suggests that antibodies to NA may be a useful therapy and that the efficacy of influenza vaccines could be enhanced by ensuring the appropriate content of NA antigen.

## INTRODUCTION

H1N1 virus entered the human population from birds in 1918. It is thought to have jumped from humans to pigs in that epoch, and it was from the pig that influenza virus was first isolated in 1931 (1); it was then isolated from humans in 1933 through infection of ferrets (2). H1N1 viruses circulated continuously in humans until 1957, when newly emerged H2N2 viruses replaced them. H1N1 virus reappeared in 1977 and continued to circulate until 2009. During this whole period, it underwent independent but continuous genetic and antigenic drift in humans and pigs. In 2009, a novel swine origin H1N1 virus reentered the human population and caused a pandemic. The accumulated sequence disparity between these independent descendants of the 1918 H1N1 virus had resulted in sufficient loss of cross-immunity to render most humans susceptible to infection by the porcine H1N1 virus in 2009.

Antibodies to the hemagglutinin (HA) and neuraminidase (NA) proteins can independently provide protection from influenza disease (3–6). The study of antibodies targeting NA has been under the shadow of the study of those against HA, although there exists an extensive amount of evidence in support of protective immunity against NA. Previous work by Schulman et al. and Kilbourne et al. showed the protective effects of anti-NA antibodies in mice and ferrets. Mice inoculated with virus or purified NA protein elicited protective immunity against NA (7, 8). The anti-NA antibodies were shown to inhibit NA activity *in vitro* and reduce virus plaque size (9). Anti-NA immunity protected mice from infection, presumably by abrogating the release of virus from infected cells. Many groups subsequently elaborated the protective effects of antibodies against NA in animal models (10–12; reviewed in references 13 to 15).

Kilbourne et al., Schild, and Couch et al. also showed that protective anti-NA antibodies are elicited in humans following natural infection (16, 17) and exposure to inactivated whole-virus vaccine (18). Current challenge studies in humans also confirm the independent protective effect of antibodies against NA (5). Finally, several groups have recently established the anti-NA antibody titers in human sera to be a correlate of protection in large clinical trials (3–5).

In contrast to a considerable literature on human monoclonal antibodies (MAbs) against HA, the majority of MAbs targeting NA described to date are from mice and rabbits, and they show relatively limited cross-reactivity. MAbs NC10 and NC41, among the first murine MAbs against NA and specific to the N9 NA, were analyzed for functional and structural characteristics (19, 20). Murine antibody CD6, which was protective against a limited range of N1-subtype viruses, including seasonal H1N1, H1N1pdm09, and avian H5N1 viruses, was found to make several contacts with adjacent NA monomers. However, this antigenic epitope underwent amino acid substitution (D451G [encoding a change of D to G at position 451]) in clade 6A H1N1pdm09 in 2012 viruses that prevented CD6 binding (12, 21).

Antibodies against NA act mainly through steric hindrance to block interaction of the active site of the enzyme with sialic acid templates, but they may also invoke Fc-dependent protective mechanisms *in vivo* (22–24). Antibody HCA-2, which was induced in rabbits by immunization with a 9-mer conserved peptide from the NA active site (residues 222 to 230), is known to bind to the active site (11, 25). This antibody reacts with a very wide range of NAs in Western blots and cross-inhibits multiple viruses of different influenza A and influenza B lineages, but only at a high concentration. HCA-2 offers only partial protection, even at the high antibody dose of 60 mg/kg of body weight, and can be affected by amino acid substitutions in the active site that lead to reduced susceptibility to NA inhibitors (11). The requirement for such a high concentration of HCA-2 is probably because it reacts with a linear epitope exposed predominantly after denaturation of NA. Thus, there is scope for potent and broadly reactive human MAbs against NA that confer better protection and could be used therapeutically.

Due to high sequence diversity in the globular head of HA, humans produced broadly reactive antibodies to the conserved stalk of HA after exposure to H1N1pdm09 virus, targeting shared epitopes in the stalks of earlier seasonal H1N1 and H1N1pdm09 viruses (26, 27). Antibodies against NA are less well studied in this context, but recently, broadly reactive anti-NA antibodies have been isolated from humans after infection (28, 29). The NA of H1N1pdm09 viruses may have reactivated B cell memory for rare epitopes shared with the N1 of earlier human seasonal viruses. The authors found that 14 to 35% of influenza A virus-specific MAbs induced by natural infection bound NA, whereas only 0 to 2% did so after vaccination. They confirmed that the NA antigen is poorly represented in many subunit vaccines and that the quality and quantity of NA in different vaccines varies (30, 31).

Despite this variability, we report a panel of anti-NA MAbs with exceptionally broad reactivity, isolated from human donors after influenza vaccination or infection. Two broadly reactive human MAbs to N1 NA, isolated from a vaccinated individual, inhibited the enzymatic activity of N1 NAs from viruses circulating in the course of the last 100 years. In addition, both MAbs cross-inhibited many N1 NAs from highly pathogenic avian influenza H5N1 viruses. The antibodies were effective prophylactics, protecting a commonly used mouse strain against the highly lethal Cambridge variant of H1N1 virus, A/PR/8/1934, and the DBA/2 mouse strain, highly susceptible to influenza, against an H1N1pdm09 virus. We also describe an antibody induced by acute H7N9 infection that cross-reacts between the human seasonal and avian N1 (group 1) and avian N9 (group 2) NAs. These exceptionally broadly reactive anti-NA MAbs offer the hope of developing vaccines that could induce them.

(This article was submitted to an online preprint archive [32].)

## RESULTS

### Antineuraminidase MAbs from human donors.

Two antibodies, AG7C and AF9C, were isolated from an adult (aged 23 years; donor C) vaccinated with 2014/15 Northern Hemisphere trivalent influenza vaccine (TIV) containing A/California/7/2009 (reassortant NYMC X-179A) (H1N1), A/Texas/50/2012 (reassortant NYMC X-223) (H3N2), and B/Massachusetts/2/2012 (reassortant NYMC BX-51B), all at 15 μg/0.5 ml (AdimFlu-S, produced by Addimmune Corporation, Taiwan) (Table 1). A third antibody, Z2B3, was isolated from a Chinese male child (donor Z) with a mild H7N9 infection in 2013; two more antibodies, Z2C2 and Z1A11, were isolated from this donor. Similarly, three more N9 MAbs were isolated from donors W and K, who were hospitalized with H7N9 virus infection (Tables 1 and 2). Antibodies to H7 HA from donors Z and K were reported previously (33).

**TABLE 1:** Data for donors and the anti-NA antibodies isolated from them

Donor	Age (yrs), gender, yr of collection	Antigen exposure	Antibody(ies) isolated	mAb specificity(ies)
Donor C	23, male, 2014	2014/15 inactivated TIV (AdimFlu-S)	AG7C, AF9C	Specific to N1 NA
Donor Z	6, male, 2013	Mild H7N9 infection	Z2B3, Z2C2, Z1A11	Cross-reactive to N1 and N9 NAs
Donor W	7, female, 2013	Severe H7N9 infection 2013	W1C7	N9 NA
Donor K	39, male, 2014	Severe H7N9 infection 2013	P17C, F4C	N9 NA, weak to N1

**TABLE 2:** Encoding-gene analysis of antibodies

MAb	Donor, age (yrs)	Genetic characteristics of^*a*^:													
		Heavy chain							Light chain						
		V gene and allele	J gene and allele	D gene and allele	V region		CDR lengths^*b*^	Sequence of aa junction	Type	V gene and allele	J gene and allele	V region		CDR lengths^*b*^	Sequence of aa junction
					% identity	No. of aa changes						% identity	No. of aa changes		
AG7C	C, 23	4-31*03 or *06	4*01 or 4*02	5-24*01	89.0	15	10.7.11	CARDLEGHTFHDW	κ	1-39*01 or 1D-39*01	2*01	88.5	17	6.3.9	CQQSHSAPYTF
AF9C	C, 23	1-69*01 or 1-69D*01	6*02	4-17*01	91.7	16	8.8.19	CARDLAPYGDRFYFHYGMDVW	κ	1-9*01	5*01	94.6	9	6.3.9	CQQLNNYPFTF
Z2B3	Z, 6	1-69*01 or 1-69D*01	6*02	5-18*01	96.5	8	8.8.25	CARDLQDTPMVDRIIGSYYYYNGLDVW	λ	2-14*01	2*01 or 3*01	96.9	8	9.3.10	CSSYTRSSSVVF
Z2C2	Z, 6	3-66*01 or *04	6*02	2-21*02	93.3	13	8.7.27	CASWSFCGGDCYPDRMQEKFHYSYGMDVW	κ	1D-12*01	4*01	95.7	9	6.3.9	CQQAYSFPLTF
Z1A11	Z, 6	1-46*01 *02 or *03	6*02	3-22*01	92.7	17	8.8.19	CARNSYYYDTDRPYYNGMDVW	κ	2-28*01 or 2D-28*01	5*01	96.9	5	11.3.9	CMQAVQTPRTF
W1C7	W, 7	3-9*01	3*02	4-17*01	99.7	0	8.8.13	CAKDVGGDYHAFDIW	κ	3-15*01	4*01	99.6	1	6.3.10	CQQYNNWPPLTF
P17C	K, 39	3-23*04	5*02	2-15*01	99.3	1	8.8.14	CAKDGRWLLGNWFDPW	λ	2-14*01	1*01	99.3	2	9.3.10	CSSYTSSSTFVF
F4C	K, 39	4-59*01	4*02	4-17*01	99.7	1	8.7.10	CARGYYGDYDYW	λ	1-40*01	2*01 or 3*01	100.0	0	9.3.11	CQSYDSSLSGVVF

a aa, amino acid.

b CDR, complementarity-determining region; CDR length, the number of amino acids in CDR1, CDR2, and CDR3, with the values for each separated by periods.

### Inhibitory breadth of anti-N1 NA MAbs against human H1N1 viruses.

**FIG 1:**
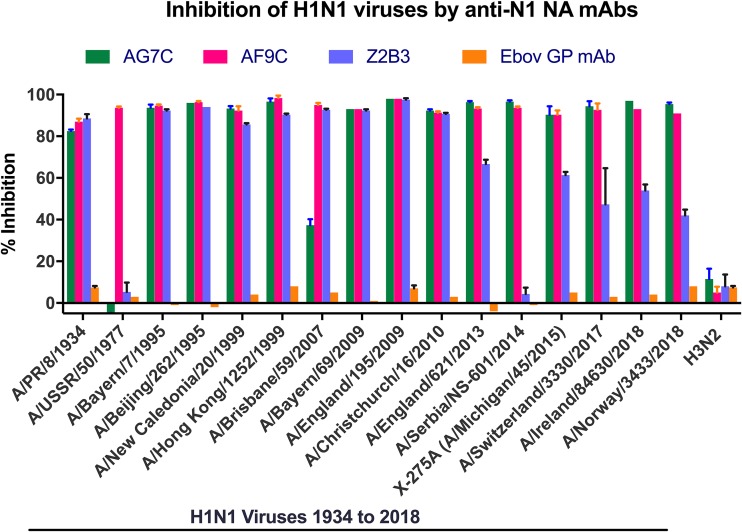
Inhibition of H1N1 viruses by MAbs targeting N1 NAs. Percentages of inhibition of activity by MAbs, at 20 μg/ml, targeting N1 NAs of the indicated viruses. An H3N2 virus (X-31) was used as a negative-control virus, and an MAb targeting Ebolavirus glycoprotein was used as a negative-control antibody. Error bars show standard deviations.

**FIG 2:**
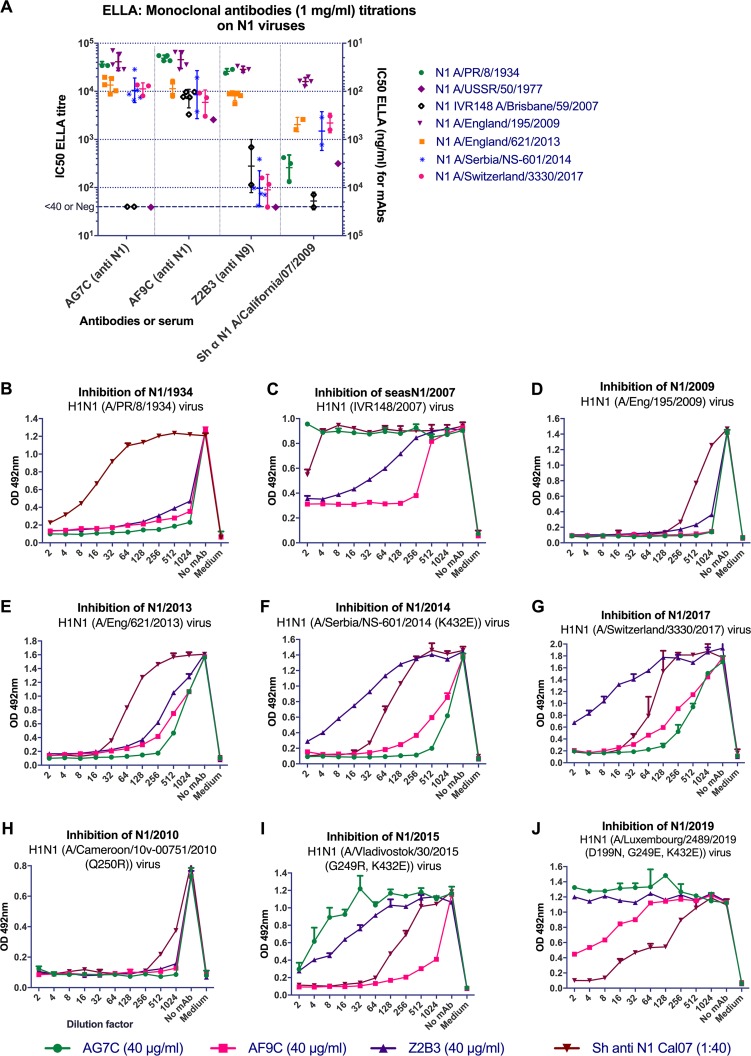
ELLA titrations of MAbs against selected H1N1 viruses. AG7C and AF9C are N1 NA-specific antibodies. Z2B3 is an N9 and N1 NA-cross-reactive antibody. Sheep anti-H1N1pdm09 N1 (A/California/07/2009) antiserum was used as a positive anti-N1 NA control. (A) ELLA IC_50_s of anti-N1 MAbs shown as titrating from 1 mg/ml on the left side of the *y* axis to compare with sheep antisera and the 50% inhibitory concentration shown as ng/ml on the right side of the *y* axis (titer of 1 = 10e6 ng/ml; inversely proportional). Each point represents an independent measurement. Geometric mean values and standard deviations are shown. (B to J) NA inhibition curves for H1N1 viruses from year 1934 to 2019. Note the reductions in titer of MAbs due to the indicated mutations: MAb Z2B3 on viruses isolated after 2014 (F, G, I, J), most likely due to K432E; MAb AG7C, likely due to G249E/R (I, J); and MAb AF9C, due to D199N (J). Experiments were done at least three times. Representative graphs are shown, with the mean value and standard deviation (*n* = 2) of each point. OD 492nm, optical density at 492 nm.

**FIG 3:**
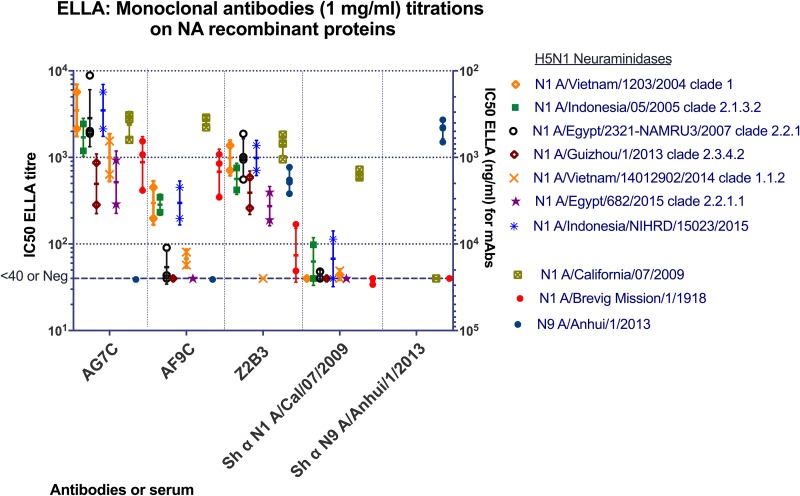
Antibodies (1 mg/ml) were titrated against recombinant NA proteins by ELLA. Sheep (Sh) antisera raised against H1N1pdm09 (A/California/07/2009) and H7N9 (A/Anhui/1/2013) viruses were used as controls. Each point represents an independent measurement. Geometric mean values and standard deviations are shown.

We focused our analysis on three MAbs, AG7C, AF9C, and Z2B3, since the other antibodies were either of limited specificity or weaker in their inhibition of NA. These three MAbs were tested for the inhibition of NA activity of H1N1 viruses isolated between 1934 and 2018, using an enzyme-linked lectin assay (ELLA) (Fig. 1 and 2), and for inhibition of the enzyme activity of the 1918 pandemic H1N1 and of avian N1 and N9 NAs as recombinant proteins (Fig. 3; Table 3).

**TABLE 3:**
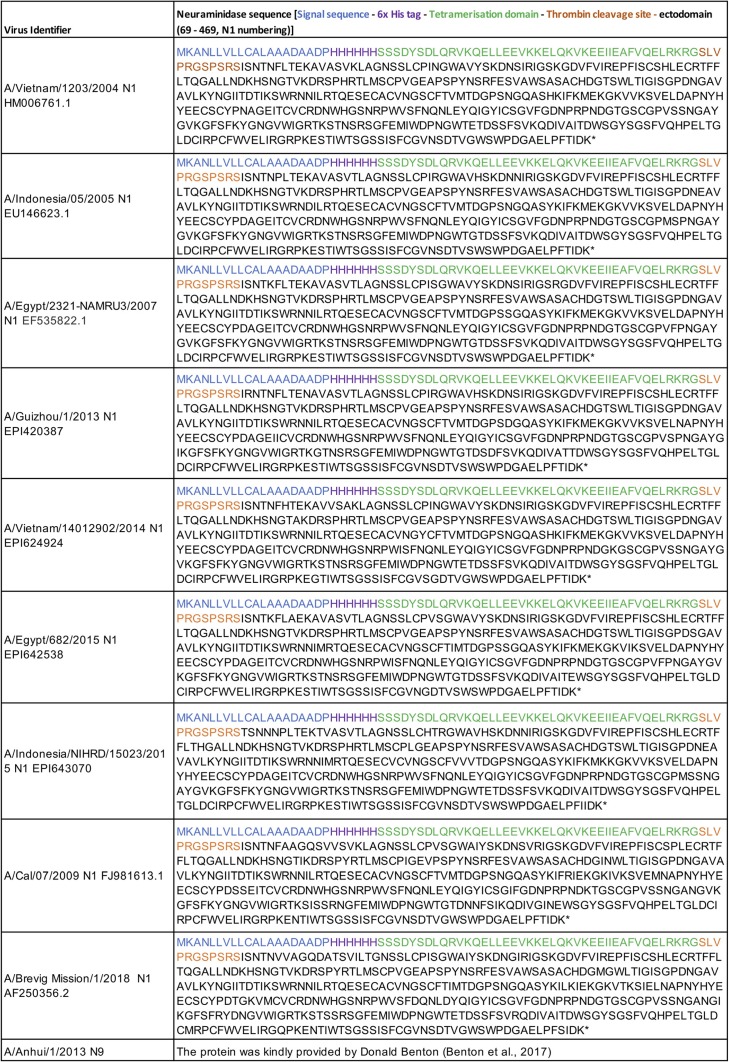
Sequences of secreted NA proteins^*a*^

a The accession number follows the virus identifier.

The MAbs were titrated by ELLA, and the concentrations required to give 50% inhibition (IC_50_) of NA activity were calculated by linear interpolation. The titers yielded by a 1-mg/ml solution were then calculated and plotted for comparison to control hyperimmune sheep antisera obtained from the National Institute for Biological Standards and Controls (NIBSC) (Fig. 2 and 3).

MAb AF9C inhibited the NA activities of all H1N1 viruses tested, which were representative of those that have circulated in humans for over 100 years (Fig. 1 and 3). MAb AG7C showed a slightly different specificity, as it weakly inhibited or failed to inhibit the NAs from A/Brisbane/59/2007 and A/USSR/90/1977 (Fig. 2). MAb Z2B3, cross-reactive with N9 NA, also showed a broad recognition of N1 NAs but again weakly inhibited A/Brisbane/59/2007 NA and failed to inhibit A/USSR/90/1977 NA (Table 1; Fig. 1 and 2). Unlike AG7C and AF9C, Z2B3 had greatly reduced activity against recent clade 6B.1 H1N1pdm09 viruses isolated after 2014 (Fig. 2).

The results in Fig. 2 show that AG7C and AF9C predominantly titrate between 1:4,000 and 1:40,000 (IC_50_s of ∼250 to 25 ng/ml) on the set of viruses shown, with the exception that AG7C fails to inhibit N1 NA from A/Brisbane/59/2007. In contrast, Z2B3 gave similar titers on A/PR/8/1934, A/England/195/2009, and A/England/621/2013 but had drastically reduced titers on A/USSR/90/1977 and the representative recent clade 6B.1 H1N1pdm09 viruses A/Serbia/NS-601/2014 and A/Switzerland/3330/2017, indicating that the genetic and associated antigenic drift in these viruses had resulted in a major alteration in the epitope recognized by Z2B3. The control hyperimmune sheep antiserum to A/California/07/2009 N1 showed limited cross-reactivity on recently drifted or older (former seasonal) viruses, with only weak activity against N1 NA from A/PR/8/1934. The sheep anti-H7N9 (A/Anhui/1/2013) antiserum contained anti-N9 NA antibodies that did not cross-react with any NAs expressed by these H1N1 viruses.

### The inhibitory activities of broadly reactive anti-N1 MAbs against NAs of avian H5N1 viruses.

To avoid handling avian influenza viruses, we titrated the MAbs for inhibition of recombinant N1 NAs from a range of H5N1 viruses isolated from infected humans, representing several HA clades from pandemic virus A/Brevig Mission/1/1918 and N9 NA from H7N9 virus A/Anhui/1/2013, produced in HEK293 cells, with N1 NA from A/California/07/2009 as a positive control (Table 3; Fig. S1 and S2 in the supplemental material).

AG7C inhibited all of the N1 NAs representing H5N1 viruses between 2004 and 2015 and the N1 NA from the 1918 pandemic virus A/Brevig Mission/1/1918. AF9C showed similar activity against N1 NAs from A/California/07/2009 and A/Brevig Mission/1918 but reacted less well with N1 NAs from H5N1 viruses (Fig. 3). Neither AG7C nor AF9C inhibited the N9 NA. In contrast, Z2B3 inhibited the H1N1pdm09 NA, the 2013 N9 NA, and most of the avian N1 NAs at moderate IC_50_s that were in general weaker than for MAb AG7C; it inhibited the 1918 N1 NA weakly. The control hyperimmune sheep antiserum against H1N1pdm09 NA showed a titer of >1:400 with A/California/7/2009 N1 NA, with minimal cross-reactivity against avian N1 NAs, 1918 N1 NA, and the 2013 N9 NA. The control sheep antiserum against N9 NA inhibited N9 but not N1 NAs.

### Anti-N9 NA MAbs cross-reactive with N1 NA.

**FIG 4:**
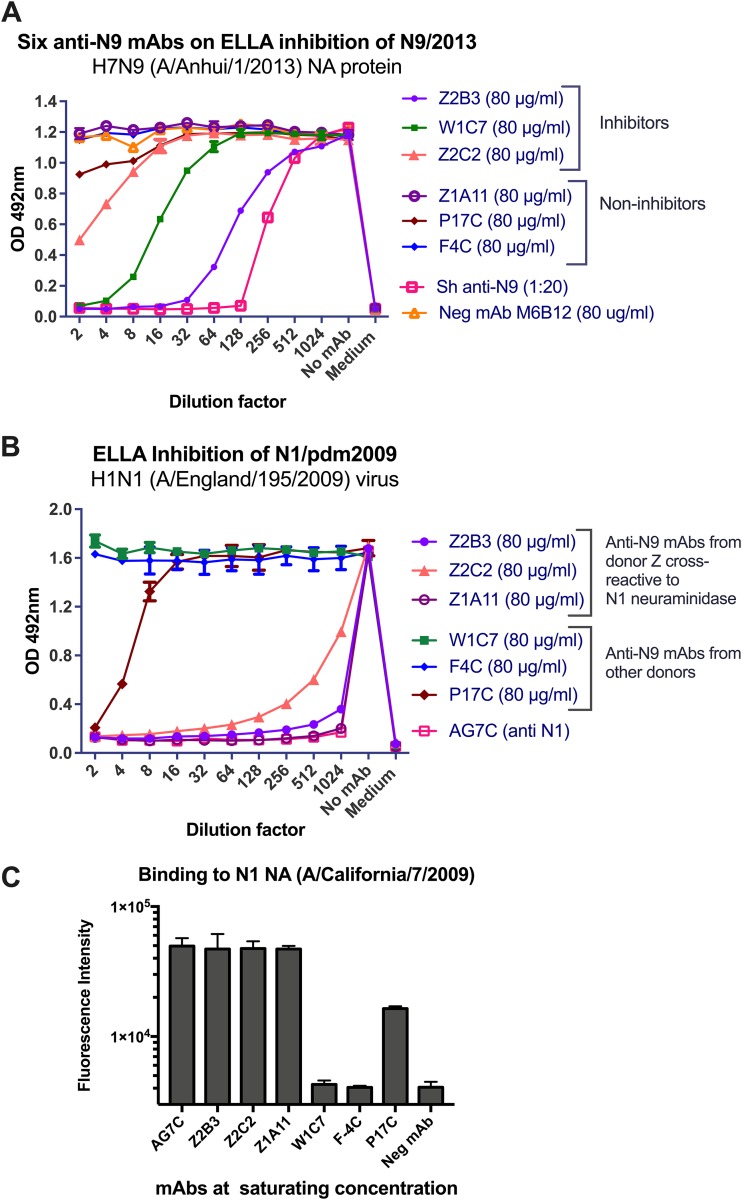
Inhibition of NA activity by MAbs isolated from donors exposed to H7N9 virus. (A) ELLA activities of six anti-N9 antibodies on N9 NA (A/Anhui/1/2013). Sheep serum raised against H7N9 virus (A/Anhui/1/2013) acts as a positive control. Anti-N2 NA MAb M6B12 was used as a negative control. Experiments were performed at least twice, and a representative graph with the mean value and standard deviation (*n* = 2) of each point is shown. (B) Cross-inhibition of N1 NA by some anti-N9 NA MAbs. Anti-N1-NA MAb (AG7C) is a positive control. Experiments were performed at least twice, and a representative graph with the mean value and standard deviation (*n* = 2) of each point is shown. (C) Binding of anti-N9 NA MAbs to H1N1 (X-179A A/California/7/2009)-infected MDCK-SIAT cells. Experiments were performed at least twice, and a representative graph with mean values and standard deviations is shown.

Among six anti-N9 NA MAbs isolated from three donors exposed to H7N9 virus and tested by ELLA, three inhibited recombinant N9 NA (Fig. 4). Two N9 NA-inhibiting MAbs were isolated from donor Z, of which Z2B3 was a strong inhibitor and Z2C2 was a weak inhibitor (Fig. 4A). All three MAbs from donor Z were cross-reactive with N1 NA (Fig. 4C) and strongly inhibited the H1N1pdm09 (A/England/195/2009) N1 NA (Fig. 4B). This suggests that 6-year-old donor Z may have made a primary antibody response to the H1N1pdm09 N1 NA and that subsequent infection with H7N9 stimulated the memory B cells to an epitope conserved between N1 and N9 NAs. Notably, Z2B3 and Z2C2 have longer heavy chain complementarity-determining region 3 (CDR3) domains than other MAbs, and although Z2B3 and AF9C are both encoded by the same variable heavy (VH) gene (VH1-69), their CDR3 amino acid sequences are significantly different.

Antibodies from donors W (W1C7) and K (P17C and F4C) were found to bind N9 NA in an indirect immunofluorescence screen (not shown). W1C7 and F4C were specific for N9 NA, and W1C7 had a weak inhibitory effect on N9 in ELLA (Fig. 4). P17C cross-reacted with N1 NA with a low level of binding and showed weak inhibition by ELLA (Fig. 4B and C).

Antibodies from donor Z have higher numbers of amino acid substitutions in the variable regions of heavy and light chains than do MAbs from other donors (Table 2). The numbers of substitutions in VH genes of MAbs Z2B3, Z2C2, and Z1A11 are 8, 13, and 17, respectively, whereas there are none, 1 and 1, respectively, in MAbs W1C7, P17C, and F4C (Table 2). This suggests that the MAbs from donor Z are of memory B cell origin, while those from donors W and K resulted from *de novo* responses to acute H7N9 infection.

### Anti-NA MAbs provide prophylactic protection *in vivo*.

**FIG 5:**
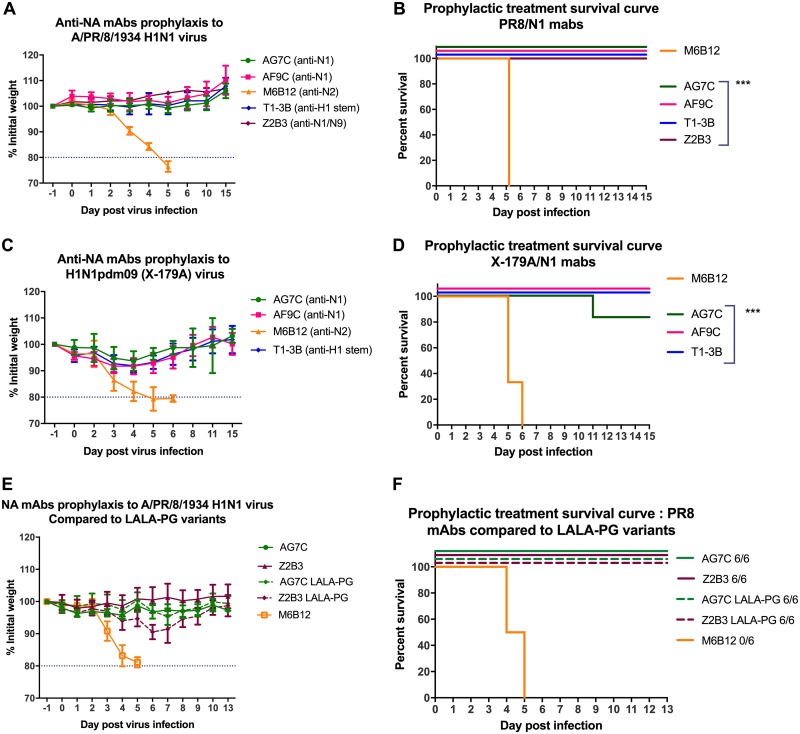
*In vivo* prophylactic protection by anti-N1 NA MAbs. Mice (*n* = 6/group) were administered AG7C and AF9C MAbs at 10 mg/kg. Weight loss following infection was measured, and ≈20% loss was considered the predefined endpoint. An anti-H1 HA MAb (T1-3B) that is cross-reactive with X-179A and A/PR/8/1934 viruses was the positive control, and an anti-N2 NA-specific MAb (M6B12) was the negative control. (A, B) Anti-N1 NA MAbs protected BALB/c female mice completely against 10^4^ TCID_50_ of A/PR/8/1934 virus, without any weight loss (*P* < 0.001). Experiments were performed at least twice, and representative data from individual experiments are shown here. (C, D) Anti-N1 NA MAbs protected DBA/2 female mice completely against a lethal dose (∼150 LD_50_) of X-179A virus (A/California/7/2009), with only 5 to 10% weight loss (*P* < 0.001). One mouse treated with AG7C relapsed on day 7 and was culled after losing ≈20% weight. (E, F) Prophylactic protection against A/PR/8/1934 virus with N1 MAbs was compared with that of their LALA-PG variants in BALB/c mice (*n* = 6). Error bars indicate standard deviations. ***, *P* < 0.001 compared to results for negative control MAb M6B12.

All three of the anti-N1 NA MAbs, AG7C, AF9C, and Z2B3, protected 100% of mice from challenge with 10^4^ median tissue culture infectious dose (TCID_50_) of A/PR/8/1934 virus (equivalent to 1,000 median lethal dose [LD_50_]) when given at a dose of 10 mg/kg 24 h before infection (*P* < 0.001) (Fig. 5A and B). They prevented any weight loss, whereas mice that received an anti-N2 NA MAb (M6B12) succumbed to ≈20% weight loss by day 5 and were humanely culled. An antibody to the H1 stem, T1-3B (34), provided a positive control for protection.

In another experiment, DBA/2 mice, which are highly susceptible to influenza infection (35), were treated with AG7C and AF9C antibodies 24 h before infection with 10^4^ TCID_50_ (equivalent to 150 LD_50_) of X-179A virus, a reassortant containing the H1N1pdm09 viral RNA from A/California/07/2009 (Fig. 5C and D). Treated mice were protected from ≈20% weight loss (*P* < 0.001), whereas mice receiving a nonspecific antibody had to be culled on day 5 or 6. One of 6 mice in the AG7C group was sacrificed on day 11 after losing ≈20% weight. In these prophylactic protection experiments, anti-NA MAbs were as protective as T1-3B, the positive-control anti-HA stalk MAb (34).

**FIG 6:**
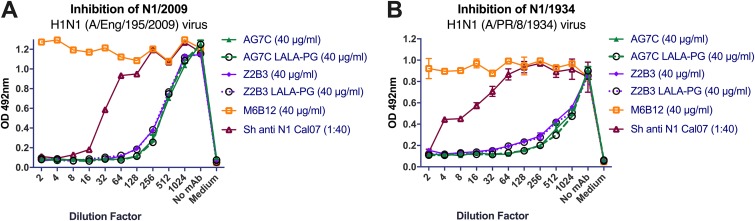
Inhibition of N1 NAs of A/England/195/2009 (A) and A/PR/8/1934 (B) by anti-N1 MAbs and their LALA-PG variants in ELLAs. Experiments were performed at least twice, and representative graphs with mean values and standard deviations are shown.

Next, we compared the prophylactic protection by N1 MAbs to that of their IgG1 L234A, L235A, and P329G (LALA-PG) variants. These substitutions abrogate Fcγ-dependent antibody-dependent cell cytotoxicity (ADCC) and complement binding and fixation (36). We confirmed that these MAbs showed no difference in their levels of inhibition of N1 NAs of X-179A and A/PR/8/1934 viruses (Fig. 6). BALB/c mice were given 10 mg/kg antibody 24 h before intranasal infection with 10^4^ TCID_50_ A/PR/8/1934 virus. We found that Fc abrogation made no difference for MAb AG7C. Both the native antibody and the LALA-PG variant protected 6/6 mice without any weight loss or clinical sign (Fig. 5E and F). However, with MAb Z2B3, there was up to 10% weight loss in mice treated with the LALA-PG variant, even though 6/6 mice were protected. This may indicate that for some antibodies to NA, Fc receptor-mediated function may contribute to the protection.

## DISCUSSION

**FIG 7:**
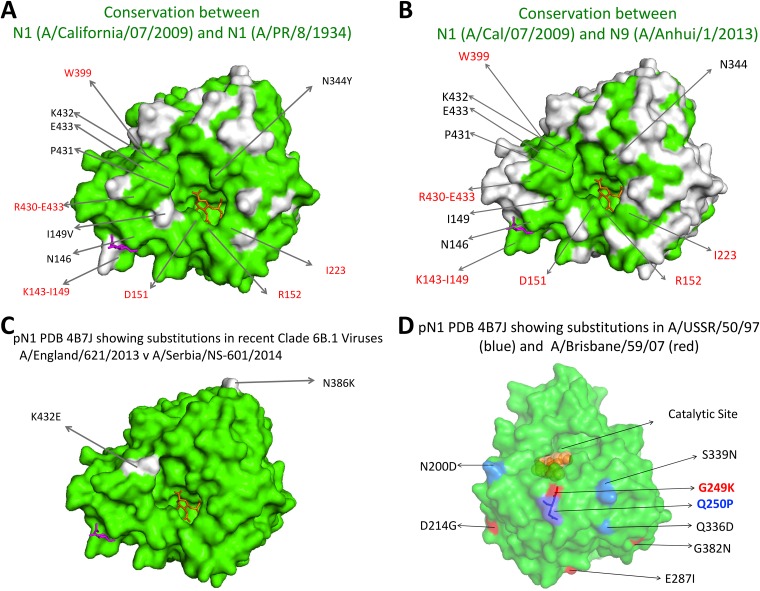
Comparisons of conserved and variable surface residues between NA subtypes. (A) Molecular surface conserved between H1N1pdm09 A/California/07/2009 and H1N1 A/PR/8/1934 (PDB ID 4B7J) is shown in green. (B) Molecular surface conserved between H1N1pdm09 (A/California/07/2009) and H7N9 (A/Anhui/1/2013) (PDB ID 4B7J) is shown in green. (C) Differences in molecular surfaces between H1N1pdm09 viruses A/England/621/2013 and A/Serbia/NS-601/2014 are shown in white. (D) Key amino acid substitutions in two H1N1 virus NAs that MAb AG7C inhibits poorly—A/USSR/50/1997 (shown in blue) and A/Brisbane/59/2007 (shown in red)—compared to NA of H1N1pdm09. These amino acid positions were inferred from the NA sequence alignment shown in Fig. S2. G249 and Q250 are likely to form part of the binding footprint of MAb AG7C. Images were generated with Pymol2 (Schrodinger LLC).

We show in this paper that broadly reactive and protective antibodies to N1 NA can be isolated from vaccinated and infected individuals, presumably due to the conservation in surface structure between N1 NAs (Fig. 7A). The two N1 subtype-specific MAbs AG7C and AF9C were isolated from the same donor, who had been vaccinated in 2014 with AdimFlu-S TIV in Taiwan. AG7C inhibits N1 NAs from H1N1 viruses isolated between 1918 and 2018. Although previous investigations of subunit vaccines have found various, usually low levels of NA antigen (14, 28, 30), in this case, there was clearly enough to induce a response.

The very broad reactivity of these MAbs with N1 NAs, covering the complete period of H1N1 virus circulation in humans, may have been induced by exposure to the significantly different NA derived from the H1N1pdm09 virus. Both MAbs show significant sequence divergence (Table 2), suggesting that they originated from a memory population which went through multiple rounds of selection in germinal centers following previous exposures to influenza. Both MAbs provided prophylactic protection to mice against the highly virulent variant of A/PR/8/1934 (the Cambridge strain) (37) and, in DBA/2 mice, against infection with H1N1pdm09 X-179A (A/California/7/2009). In an earlier paper, Chen et al. described similar anti-N1 NA antibodies that reacted with viruses spanning the period from 1918 to 2009 (28).

The third antibody, Z2B3, was isolated from a child who experienced a mild infection with H7N9 virus in 2013. It was unusual in being cross-reactive with group 1 (N1) and group 2 (N9) NAs (Fig. S1 in the supplemental material). Two similar antibodies were isolated from this donor, both of which inhibited N1 NA with some level of cross-reaction with N9 NA (Fig. 4), which we interpret to imply that they were selected from a subpopulation of memory cells induced previously by N1 NA. Examination of the structure of the N1 and N9 NAs reveals a region of conserved surface around and within the active site of the enzyme as a possible binding site for Z2B3 (Fig. 6B).

MAb Z2B3 showed good reactivity with the H1N1pdm09 virus A/England/621/2013 but poor reactivity with a later clade 6B virus, A/Serbia/NS-601/2014. These two viruses showed nonconservative amino acid substitutions of only N386K and K432E in the head of NA (Fig. 7C). The former site has a similar substitution in the N9 NA that Z2B3 recognizes, which suggests that K432 is within the footprint of MAb Z2B3. K432 falls within a known epitope recognized by anti-N9 NA antibodies (19, 38). The crystal structure of an N9 NA-MAb complex, N9-NC10, involved a contact between D56 of the antibody H chain and K432 of N9 NA (sequence, GRPKEDK; PDB identifier [ID] 1NMB).

K432 was conserved prior to 2013 but underwent a K432E substitution in 2014, which became dominant thereafter. We suggest that N1 NA has been under strong evolutionary pressure from broadly cross-reactive antibodies induced by the H1N1pdm09 NA that were selected from memory B cells raised against NA(s) of earlier virus(es). Just as the conserved stalk of HA has shown a capacity for evolution under pressure from antibody selection (39), the NA may similarly be forced to drift antigenically by broadly cross-reactive antibodies induced by the H1N1pdm09 viruses (40).

With this in mind, we examined the region of the NA surface recognized by broadly reactive antibodies described by Chen et al. and Gao et al. that inhibited or bound N1 NAs of viruses isolated between 1918 and 2009 but not clade 6B H1N1pdm09 viruses (28, 40). Some of these antibodies lost binding to N1 NAs due to substitutions in a set of site-specific mutants (21, 40). Many of these antibodies also did not inhibit A/Brisbane/59/2007. MAb AG7C showed a similar reactivity profile and may have been affected by substitutions G249K and Q250P, which are common to the nonreactive NAs. These residues are exposed on the periphery of the catalytic site (Fig. 7D). The preceding residue N248 was replaced (N248D) in the H1N1pdm09 viruses isolated after 2009, which caused a loss of recognition by some MAbs described by these groups. However, this substitution is tolerated by MAb AG7C. There are rare natural isolates that have substitutes for these residues (G249E/R and Q250R), indicating that even the broadly reactive MAbs can be thwarted by virus antigenic drift (Fig. S2). MAb AG7C exhibited significant reductions of inhibition titers against viruses with G249E/R substitutions, confirming that these residues are parts of an epitope (Fig. 2I and J). However, it does not seem to be affected by the Q250R substitution (Fig. 2H). Eleven of 1,944 H1N1pdm09 viruses sampled between 2008 and September 2019 had acquired the G249E/R substitution (https://www.nextstrain.org). Substitutions for Q250 are rare; Q250L was seen in 1/1,944 viruses analyzed.

Similarly, the comparison of NA sequences between A/Switzerland/3330/2017 (inhibited strongly by AF9C) and A/Luxembourg/2489/2019 (inhibited weakly) implicated the substitution D199N in the loss of recognition and, therefore, D199 as part of the epitope recognized by AF9C (Fig. S2). Seven of 2,056 NA sequences from H1N1pdm09 viruses detected between 2013 and 2019 had N1 D199N/A substitutions. Further structural work to define the epitopes recognized by Z2B3, AG7C, and AF9C is in progress.

We found that the broadly reactive human IgG1 anti-N1 MAb AG7C did not require Fc engagement for complete protection of mice. In contrast, the LALA-PG variant of MAb Z2B3 provided protection to 100% of animals but with some weight loss, unlike the unmutated IgG. This contrasts with a broadly reactive MAb (MAb 3C05) containing human variable regions linked with murine IgG2a Fc, for which protection was abrogated by the Fc mutant DA265 (23). Some NA MAbs are known to protect *in vivo* via Fc-mediated functions even in the absence of neuraminidase activity (41). However, we have not tested noninhibiting MAbs for *in vivo* protection in this study.

It has become clear that exposure to viruses that differ significantly from those circulating can select responses to epitopes in both HA and NA that are shared between the incoming virus and the seasonal viruses in circulation, derived from the memory B cell population (42, 43). While antibodies against new epitopes can also be generated, even in the elderly (33), it appears that they are initially at a disadvantage but may overtake and become dominant with time (44, 45). It would be wise to assume that all of these epitopes, both new and conserved, can drift under pressure from antibody selection. The inevitable implication is that updating influenza vaccines may have to continue, but broadening the memory B cell population by vaccination with as wide a range of group 1 and 2 HAs and NAs as possible (46) might be a logical way of preparing the ground for a strong response to an unknown future pandemic virus.

## MATERIALS AND METHODS

### Media, reagents, and tissue culture.

MDCK-SIAT1 cells and adherent 293T cells (ECACC) were grown in Dulbecco modified Eagle medium (DMEM) (product number D5796; Sigma) supplemented with 10% (vol/vol) fetal calf serum (product number F9665; Sigma), 2 mM glutamine, 100 U/ml penicillin, and 100 μg/ml streptomycin (all from Sigma, UK). 293-F suspension cells were grown in FreeStyle 293-F expression medium (12338-018; Life Technologies) on a shaker incubator. Cells were grown at 37°C, 5% CO_2_ in a humidified incubator. Viruses were diluted and grown in virus growth medium (VGM), which is DMEM with 0.1% bovine serum albumin (product number A0336; Sigma), 10 mM HEPES, and glutamine, penicillin, and streptomycin as in D10 medium.

### Influenza viruses and control sera.

H1N1 viruses from the years 1977 to 2019 and H3N2 viruses were obtained from the Worldwide Influenza Centre (WIC) at The Crick Institute (London, UK). Other reassortant viruses and control sheep sera were obtained from the National Institute for Biological Standards and Controls (NIBSC), UK.

### Ethics and study approval.

The study was performed in compliance with good clinical practice guidelines and the Declaration of Helsinki. The protocol was approved by the Research and Ethics Committees of Chang Gung Memorial Hospital, Beijing Ditan Hospital, and the Weatherall Institute of Molecular Medicine. All subjects provided written informed consent. A list of donors with their details and isolated antibodies is in Table 1.

### Isolation of human MAbs.

MAbs were isolated from individual humans who either received seasonal influenza vaccine or were naturally infected with H7N9 virus in China or Taiwan. Peripheral blood mononuclear cells (PBMC) were collected from individual donors either a week after vaccination against influenza or from confirmed influenza virus-infected cases 10 days after onset of clinical symptoms. Antibodies were isolated from PBMC using single-cell isolation and cloning methods as described in detail previously (34, 47–49). Briefly, plasmablasts in PBMC were stained (CD3^−^, CD19^+^, CD20^low/−^, CD27^high^, and CD38^high^) and sorted as single cells using flow cytometry. mRNA from single plasmablasts was reverse transcribed to DNA, and VH and Vκ/λ genes were amplified using gene-specific primers and then cloned into expression vectors containing IgG1 heavy and Vκ and Vλ constant regions. Heavy and light chain plasmids were cotransfected into 293T or ExpiCHO cells (catalog number A29133; Life Technologies) for antibody expression.

### LALA-PG antibody variants.

The L234A, L235A, and P329G (LALA-PG) amino acid substitutions (36) that abrogate Fc-mediated functions were engineered into the human IgG1 Fc regions of antibodies AG7C and Z3B2 by standard procedures and confirmed by sequencing.

### Antibody screening.

MAbs were initially screened for binding to MDCK-SIAT1 cells infected with either H1N1 or H3N2 virus and for lack of binding to HA protein expressed in stably transfected MDCK-SIAT1 cells. Binding to NA was confirmed by immunoprecipitation with infected cells or binding to 293T cells transfected with the NA gene of interest.

### Production of NA proteins.

Tetrameric neuraminidase proteins were expressed from constructs based on the design of Xu et al. (50). In our version, the signal sequence from A/PR/8/1934 HA was followed by a human vasodilator-stimulated phosphoprotein (VASP) tetramerization domain and thrombin site, followed by amino acids 69 to 469 of the NA sequence (N1 numbering) (Table 3). Sequences were synthesized as human-codon-optimized cDNAs by Geneart and cloned into pCDNA3.1/− for transfection. HEK293F cells were transiently transfected using polyethyleneimine (PEI)-based PEIpro as a transfection reagent. Protein supernatant harvested 5 to 7 days posttransfection was titrated for NA activity in an enzyme-linked lectin assay (ELLA) and stored in aliquots at −80°C.

N9 NA protein (A/Anhui/1/13) was kindly provided by Donald Benton (The Francis Crick Institute) (51). The expression construct consisted of ectodomain residues 75 to 465 with an N-terminal 6× His tag, a human VASP tetramerization domain (50), and a TEV (tobacco etch virus) cleavage site under the control of a promoter with a gp67 secretion signal peptide. The protein was expressed in Sf9 insect cells using a recombinant baculovirus system (Life Technologies). The protein was purified on a cobalt resin column and further purified by gel filtration to ensure the removal of monomeric and aggregated protein.

For antibody inhibition measurements, a dilution of the NA-containing supernatant that had just reached plateau activity in the ELLA was chosen. The sequences of all the constructs and their accession numbers are shown in Table 3.

### ELLA for NA inhibition.

The ELLA was adapted from the methods described by Schulman et al. (7) and Sandbulte et al. (52). This assay detects the inhibition of NA’s enzymatic activity, the cleavage of sialic acid, by anti-NA antibodies. Viruses or recombinant NA proteins were used as the source of NA. Virus growth medium was used to dilute antibodies and viruses. A Nunc immunoassay enzyme-linked immunosorbent assay (ELISA) plate (catalog number 439454; Thermo Scientific) was plated overnight with 25 μg/ml fetuin (product number F3385; Sigma). Twofold serial dilutions of sera or MAbs performed in duplicates were incubated together with a fixed amount of titrated NA source. Column 11 of a plate was used for an NA-source-only control, and column 12 was used for a medium-only control. After 2 h of incubation, antibody-NA mixtures were transferred to the phosphate-buffered saline (PBS)-washed fetuin plate and incubated for 18 to 20 h at 37°C, buffered by CO_2_ as for tissue culture. On the next day, the contents of the plate were discarded, and the plate washed 4 times with PBS. Horseradish peroxidase-conjugated peanut agglutinin (PNA-HRP) (product number L7759; Sigma) at 1 μg/ml was added to the wells. PNA binds to the exposed galactose after cleavage of sialic acid by NA. After 1 h of incubation and washing with PBS, the signal was developed by adding OPD (*o*-phenylenediamine dihydrochloride) solution (product number P9187; Sigma), and the reaction was stopped after 5 to 15 min using 1 M H_2_SO_4_. Absorbance was read at 492 nm in a Clariostar plate reader (BMG Labtech).

The antibodies were titrated by doubling dilution, and the concentrations required to give 50% inhibition (IC_50_) of NA activity were calculated by linear interpolation. For comparison with the results for the positive-control sheep sera, titers of antibodies, with the starting concentration transformed to 1 mg/ml, were compared with the serum titers in the same graphs.

### *In vivo* prophylaxis protection.

All animal procedures were approved by an internal University of Oxford Ethics Committee and the United Kingdom Home Office. The experiments were carried out in accordance with the *Guide for the Care and Use of Laboratory Animals* (53), the recommendations of the Institute for Laboratory Animal Research, and Association for Assessment and Accreditation of Laboratory Animal Care International standards. The principle of the three Rs (replacement, reduction, and refinement) was applied in designing the experiments.

Mice used in protection studies, DBA/2OlaHsd mice (*n* = 6/group) for X-179A virus and BALB/cOlaHsd mice (*n* = 6/group) for PR8 virus, were purchased from Envigo, United Kingdom, and housed in individually vented cages in a special unit for infectious diseases. Mice were anesthetized with isofluorane (Abott), and 50 μl of virus was administered intranasally 24 h after the intraperitoneal administration of 10 mg/kg antibody (500 μl). Mice were observed and weighed regularly. Mice with weight loss of ≈20% or morbid clinical scores were euthanized by raising the concentration of CO_2_. Nonspecific IgG antibody was used as a negative control. Known HA-specific antibodies were used as positive controls. Mice were infected intranasally with lethal doses of viruses X-179A (150 LD_50_, 10^4^ TCID_50_) and PR8 (1,000 LD_50_, 10^4^ TCID_50_).

### Sequence analysis.

Amino acid substitutions in H1N1pdm09 viruses were analyzed by downloading sequences from the EpiFlu database of the Global Initiative on Sharing All Influenza Data (GISAID) or on https://www.NextStrain.org. The viruses were randomized for geography and year during analysis, and sequence alignment was done using BioEdit version 7. The sequences of all viruses used experimentally were determined/confirmed at the WIC.

### Data and statistical analysis.

Graphs were generated using GraphPad Prism (version 9) and Microsoft Excel 2010. Protein structures were viewed using Pymol2 (Schrodinger, LLC).

The ELLA titers were expressed as half-maximal inhibitory concentrations (IC_50_, the midpoint between negative and plateau positive controls) derived by linear interpolation from neighboring points in the titration curve. Kaplan-Maier tests were performed to analyze the differences in mortality between experimental- and control-group mice. *P* values of <0.05 were considered to show a significant statistical difference.

## Supplementary Material

Supplemental file 1
