# Supplementary material for: Broadly Inhibiting Antineuraminidase Monoclonal Antibodies Induced by Trivalent Influenza Vaccine and H7N9 Infection in Humans
Source: J Virol. 2020 Jan 31;94(4):e01182-19. doi: 10.1128/JVI.01182-19 (PMC6997757; doi:10.1128/JVI.01182-19)
Supplement: Supplemental file 1 [file JVI.01182-19-s0001.pdf]

Supplemental Data

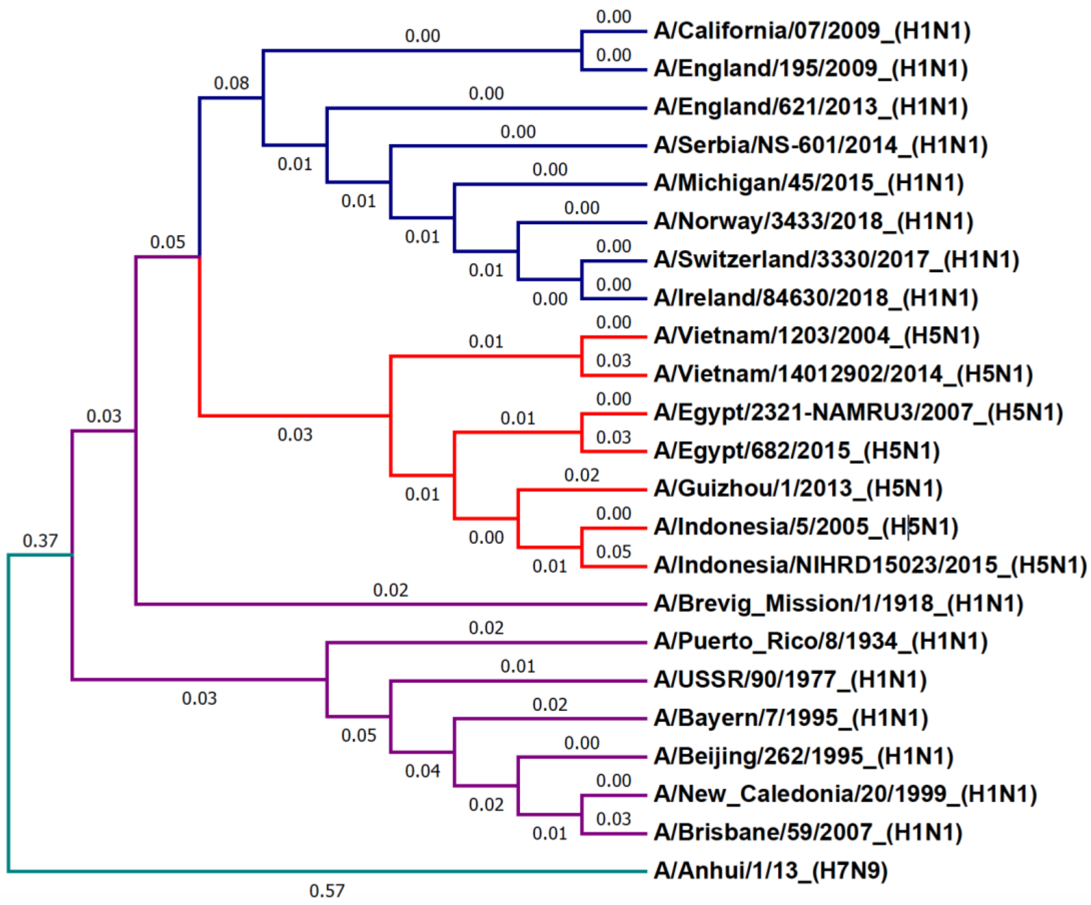

Figure S1. The phylogenetic tree of selected N1 and N9 neuraminidases used in this paper. The values on branches shows the evolutionary distances between neuraminidases. Made using MEGA7 software, muscle alignment and neighbour-joining tree settings.

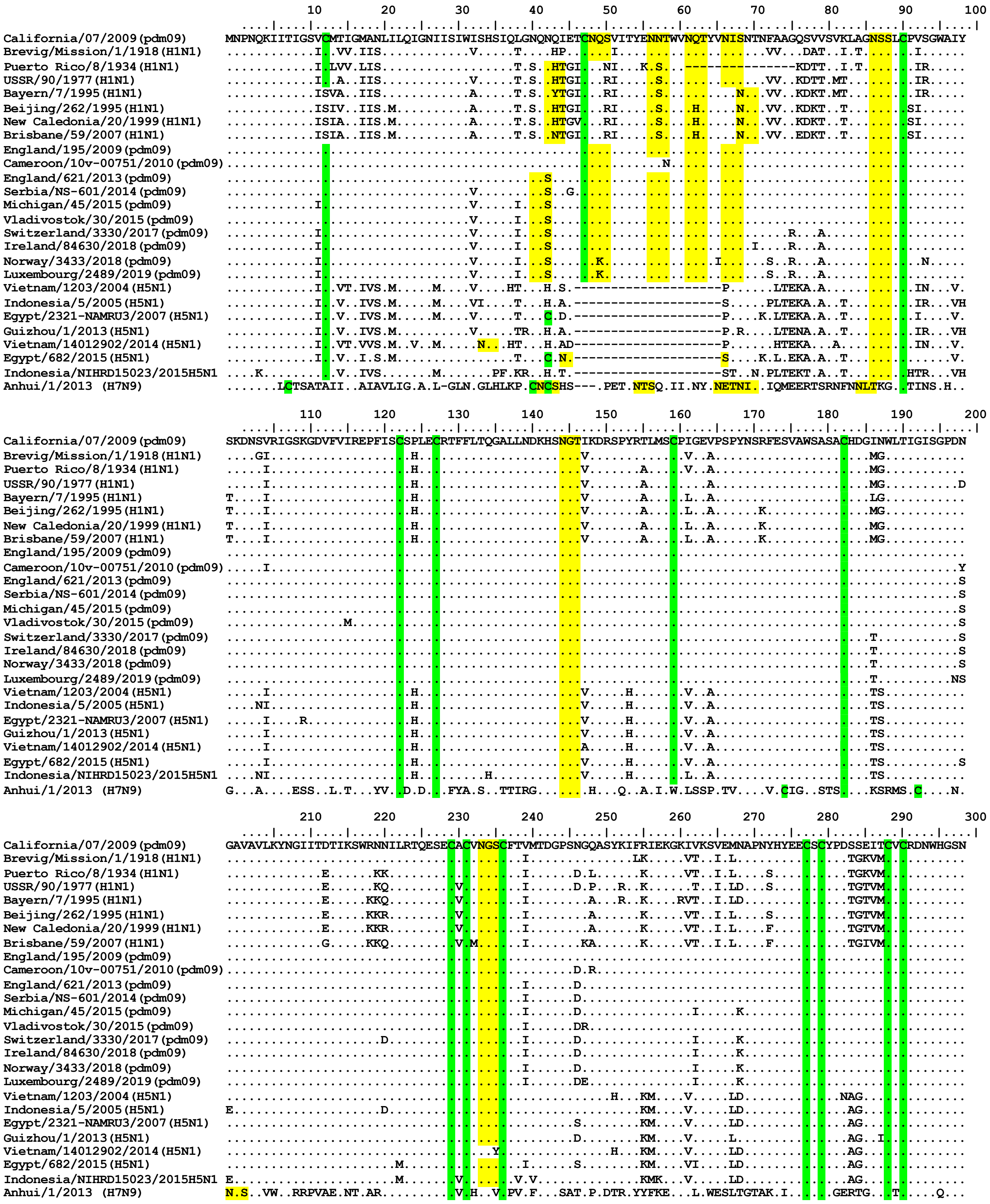
10 20 30 40 50 60 70 80 90 100
California/07/2009(pdm09) MNPNQKIITIGSVCMTIGMANLILQIGNIISIWISHSIQLGNQNQIETCNQSVITYENNTWVNQTYVNISNTNFAAGQSVVSVKLAGNSSLCPVSGWAIY
Brevig/Mission/1/1918(H1N1)
Puerto Rico/8/1934(H1N1)
USSR/90/1977(H1N1)
Bayern/7/1995(H1N1)
Beijing/262/1995(H1N1)
New Caledonia/20/1999(H1N1)
Brisbane/59/2007(H1N1)
England/195/2009(pdm09)
Cameroon/10v-00751/2010(pdm09)
England/621/2013(pdm09)
Serbia/NS-601/2014(pdm09)
Michigan/45/2015(pdm09)
Vladivostok/30/2015(pdm09)
Switzerland/3330/2017(pdm09)
Ireland/84630/2018(pdm09)
Norway/3433/2018(pdm09)
Luxembourg/2489/2019(pdm09)
Vietnam/1203/2004(H5N1)
Indonesia/5/2005(H5N1)
Egypt/2321-NAMRU3/2007(H5N1)
Guizhou/1/2013(H5N1)
Vietnam/14012902/2014(H5N1)
Egypt/682/2015(H5N1)
Indonesia/NIHRD15023/2015H5N1
Anhui/1/2013 (H7N9)
110 120 130 140 150 160 170 180 190 200
California/07/2009(pdm09) SKDNSVRIGSKGDVFVIREPFISCSPLECRTFFLTQGALLNDKHSNGTIKDRSPYRTLMSCPIGEVPSPYNSRFESVAWSASACHDGINWLTIGISGPDN
210 220 230 240 250 260 270 280 290 300
California/07/2009(pdm09) GAVAVLKYNGIITDTIKSWRNNILRTQESECACVNGSCFTVMTDGPSNGQASYKIFRIEKGKIVKSVEMNAPNYHYEECSCYPDSSEITCVCRDNWHGSN

```
                                                 310       320       330       340       350       360       370       380       390       400
                                        ....|....|....|....|....|....|....|....|....|....|....|....|....|....|....|....|....|....|....|....|
California/07/2009(pdm09)               RPWVSFNQ-NLEYQIGYICSGIFGDNPRPND-KTGSCG-PVSSNGANGVKGFSFKYGNGVWIGRTKSISSRNGFEMIWDPNGWTGTDNNFSIKQDIVGIN
Brevig/Mission/1/1918(H1N1)             ......D.-..D.........V.........-G.....-.........I.....R.D..........T...S............E..SS..VR....A.T
Puerto Rico/8/1934(H1N1)                ......D.-..D.........V.......E.-G.....-..YVD.........YR............H...H............E..SK..VR..V.AMT
USSR/90/1977(H1N1)                      ........-..D.........V.......K.-GK...D-..NVD..D......YR............N...K............D..S..LV...V.AMT
Bayern/7/1995(H1N1)                     ........-..D.........V.......K.-GE...N-..TVD.........YR............NRL.K............D..SD..M...V.AMT
Beijing/262/1995(H1N1)                  ........-..D.........V.......K.-GE...N-..TVD..D......YR............NRL.K............D..SD..V...V.AMT
New Caledonia/20/1999(H1N1)             ........-..D.........V.......K.-GE...N-..TVD..D......Y.............NRL.K............D..SD..V...V.A.T
Brisbane/59/2007(H1N1)                  ........-..D.........V.......E.-GE...N-..TVD.........Y..D..........NRL.K............N..SD..V...V.A.T
England/195/2009(pdm09)                 ........-......................-......-.............................................................
Cameroon/10v-00751/2010(pdm09)          ........-......................-......-.............................................................
England/621/2013(pdm09)                 ........-......................-......-................................K............................
Serbia/NS-601/2014(pdm09)               ........-............V.........-......-................................K................K...........
Michigan/45/2015(pdm09)                 ........-.....M......V.........-......-................................K................K...........
Vladivostok/30/2015(pdm09)              ........-............V.........-......-..L.........................T...K................K...........
Switzerland/3330/2017(pdm09)            ........-.....M......V.........-......-................................K................K...........
Ireland/84630/2018(pdm09)               ........-.....M......V.........-......-............................T...K................K...........
Norway/3433/2018(pdm09)                 ........-.....M......V.........-......-................................K................K...........
Luxembourg/2489/2019(pdm09)             ........-.....M......V.........-......-................................K................K..K....L...
Vietnam/1203/2004(H5N1)                 ........-............V.........-G.....-.......Y....................TN..S............E..SS..V.....A.T
Indonesia/5/2005(H5N1)                  ........-............V.........-G.....-.M.P...Y....................TN..S...............SS..V.....A.T
Egypt/2321-NAMRU3/2007(H5N1)            ........-............V.........-G.....-..FP...Y....................TN..S...............SS..V.....A.T
Guizhou/1/2013(H5N1)                    ........-............V.........-G.....-...P...Y.I.................GTN..S...............SD..V.....ATT
Vietnam/14012902/2014(H5N1)             ...I....-............V.........-GK....-.......Y....................TN..S............E..SS..V.....A.T
Egypt/682/2015(H5N1)                    ...I....-............V.........-G.....-..FP...Y....................TN..S...............SS..V.....A.T
Indonesia/NIHRD15023/2015H5N1           ........-............V.........-G.....-.M.....Y....................TN..S............E..SS..V.....A.T
Anhui/1/2013 (H7N9)                     ..VIQIDPVAMTHTSQ....PVLT.......PNI.K.ND.YPG.NN.......YLD.ANT.L...I.TA..S.Y..LKV..AL.DDRSKPIQG.T..LNA

                                                 410       420       430       440       450       460       470
                                        ....|....|....|....|....|....|....|....|....|....|....|....|....|....|...
California/07/2009(pdm09)               EWSGYSGSFVQHPELTGLDCIRPCFWVELIRGRPKEN-TIWTSGSSISFCGVNSDTVGWSWPDGAELPFTIDK
Brevig/Mission/1/1918(H1N1)             D...................M...........Q....-...............................S...
Puerto Rico/8/1934(H1N1)                D...................M...............K-.....A.............D...........S...
USSR/90/1977(H1N1)                      D...................M.............R.KT...................N...............
Bayern/7/1995(H1N1)                     D...................M........V..L.R..T................E.AN...............
Beijing/262/1995(H1N1)                  D............................V....R..T..................AN...............
New Caledonia/20/1999(H1N1)             D............................V..L.R..T..................AN...............
Brisbane/59/2007(H1N1)                  D............................V..L.R..T..................AN...............
England/195/2009(pdm09)                 .....................................-...................................
Cameroon/10v-00751/2010(pdm09)          .....................................-.......I...........................
England/621/2013(pdm09)                 .....................................-...................................
Serbia/NS-601/2014(pdm09)               ..................................E..-...................................
Michigan/45/2015(pdm09)                 ..................................E..-...................................
Vladivostok/30/2015(pdm09)              ..................................E..-...................................
Switzerland/3330/2017(pdm09)            ..................N...............E..-..............D....................
Ireland/84630/2018(pdm09)               ..................N...............E..-..............D....................
Norway/3433/2018(pdm09)                 ..................N...............E..-..............D..I.................
Luxembourg/2489/2019(pdm09)             ..................N...............E..-..............D..I.................
Vietnam/1203/2004(H5N1)                 D...................................S-...................................
Indonesia/5/2005(H5N1)                  D...................................S-...................S...............
Egypt/2321-NAMRU3/2007(H5N1)            D...................................S-...................S...............
Guizhou/1/2013(H5N1)                    D...................................S-...................S...............
Vietnam/14012902/2014(H5N1)             D...................................G-..............SG...................
Egypt/682/2015(H5N1)                    ....................................S-...............G...S...............
Indonesia/NIHRD15023/2015H5N1           D...................................S-...................S...........I...
Anhui/1/2013 (H7N9)                     D........MDY--WAEG..Y.A..Y..........DKVW...N.IV.M.SSTEFLGQ.N.....KIEYFL--
```

Figure S2. Amino acid sequence alignment of the neuraminidases used in this paper. The numbering is not official NA numbering and is only for alignment purpose. The alignment was done using BioEdit software. Cysteine residues are highlighted in green and potential N-linked glycosylation sequons (NXS/T) in yellow.
